# 619. Current State of Infectious Diseases Pharmacist OPAT/COpAT Practice in the United States

**DOI:** 10.1093/ofid/ofab466.817

**Published:** 2021-12-04

**Authors:** Christina G Rivera, Keenan L Ryan, Kristin Mara, Monica V Mahoney, Monica V Mahoney

**Affiliations:** 1 Mayo Clinic, Rochester, Minnesota; 2 University of New Mexico Hospitals, Albuquerque, New Mexico; 3 Beth Israel Deaconess Medical Center, Boston, Massachusetts

## Abstract

**Background:**

Outpatient parenteral antimicrobial therapy (OPAT) is the process of administering intravenous (IV) antimicrobials outside the acute inpatient setting. Oral antimicrobials for complex infections are referred to as complex outpatient antimicrobial therapy (COpAT). OPAT/COpAT programs are expanding, as are the opportunities for clinical Infectious Diseases (ID) pharmacists (RPHs) involvement. The current state of clinical (non-dispensing) role and the functions being performed by RPHs in OPAT/COpAT is unknown.

**Methods:**

To define the current state of OPAT/COpAT pharmacy practice across the United States (US), specifically the clinical functions performed by RPHs, design of RPH involved OPAT/COpAT clinics, and compare training of RPHs who practice in OPAT/COpAT to ID RPHs who do not, a survey of a possible 31 questions was emailed to the American College of Clinical Pharmacists (ACCP) Infectious Diseases Practice and Research Network (PRN) email list. Results were focused on US-based respondents.

**Results:**

Eighty-seven RPHs responded with 27 practicing in OPAT/COpAT. Training background did not differ between groups. Programs with an OPAT/COpAT RPH were more likely to have a formal OPAT team compared to those without an OPAT/COpAT RPH (p < 0.001). OPAT/COpAT RPHs were early in their careers, with roughly half practicing < 5 years in ID, and 66.7% practicing < 5 years in OPAT/COpAT. Most OPAT/COpAT RPHs (66.7%) practiced at an academic medical center with a median full time equivalent (FTE) of 1 RPH. Most (63%) utilized a collaborative practice agreement and 81.5% shared job functions with other ID RPH roles, most commonly antimicrobial stewardship. Few (28%) OPAT/COpAT programs involved a dispensing component. The average daily census was 42 patients followed by an OPAT/COpAT RPH. There was wide variability in the types of tasks ID RPH performed in OPAT/COpAT, the three most important tasks are listed in Figure 1.

OPAT Pharmacists Task Ranking by Importance

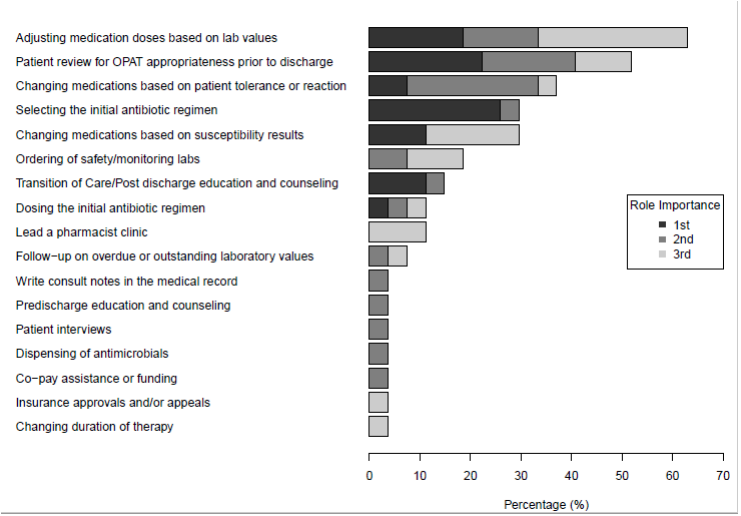

There was wide variability in the types of tasks ID pharmacist performed in OPAT/COpAT. The most OPAT/COpAT pharmacists responded that adjusting medications based on lab values was in their top 3 most important clinical tasks. When ranking the top three most important tasks, selecting the initial OPAT/COpAT regimen was ranked first most often, followed by review of review of OPAT appropriateness for discharge, then adjusting medications based on lab values.

**Conclusion:**

This is the largest known survey of OPAT/COpAT RPHs. RPH involvement in OPAT/COpAT in the US is an emerging trend with wide variability in program structure. Tasks performed by OPAT/COpAT RPHs varied significantly; however, OPAT/COpAT RPH respondents’ functions are largely clinical in nature.

**Disclosures:**

**All Authors**: No reported disclosures

